# Current News and Opinion

**Published:** 1883-01

**Authors:** 


### Current News and Opinion.

THE NEW YORK MEDICO-LEGAL SOCIETY.—At the regular stated meeting, held at Mott Memorial Hall December 6, 1882, officers were elected for the ensuing year as follows: Clark Bell, Esq., President; Prof. R. O. Doremus, 1st Vice President; Hon. D. C. Calvin, 2d Vice President; L. P. Holmes, Esq., Secretary; G. R. Hawes, Esq., Assistant Secretary; J. Shrady, Esq., Treasurer; R. S. Guernsey, Esq., Librarian; A. H. Smith, M. D., Curator and Pathologist; M. Ellinger, Esq., Corresponding Secretary; Prof. C. A. Doremus, Chemist; E. H. M. Sell, M. D., and B. A. Willis, Esq., Trustees; A. G. Hull, Esq., and M. H. Henry, M. D., Members of the Permanent Commission. The papers of the evening were by O. W. Wight, M. D., of Detroit, entitled: "What is Expert Testimony, and Who are Experts?" and by E. Sanders, M. D., entitled "Our Coroner System: Shall it Continue to Exist?" Submitted but not read.

THE BROOKLYN DENTAL SOCIETY.—At the last monthly meeting of this Society, which was held at Dr. Thompson's house, corner of Adelphi and Spring avenue, a highly interesting discussion took place upon one of the live subjects of the day, viz., Germ Caries. An able paper by Dr. Stockwell, entitled, "Dental Caries—Acids or Germs, Which?" was read to the Society by Dr. Cook, after which the President, Dr. Brockaway, called upon Dr. F. Y. Clark, of New York, to address the meeting, which it was gratifying to observe was so largely and influentially attended. Dr. Clark proceeded to read a series of extracts from former papers of his own, and then gave a general and admirable explanation of the subject, which was further taken up and discussed by Doctors Wilson, Cook, Johnson and others. Finally a resolution was unanimously passed requesting Dr. Clark to resume the subject at the next monthly meeting, which is to be held on the 8th of January at the house of Dr. Cook, No. 133 Pacific avenue, when Dr. Stockwell is to be specially invited to attend. After the adjournment of the Society, Dr. Thompson gave a handsome "spread" to the members present.

Frozen Food.—The *Medical Times and Gazette* says it is not generally known that some millions of the human race subsist for one-third of the year on frozen food, not only meat and fish, but also butter, game, milk, etc. We do not refer to the Esquimaux or Samoyedes, but to people living in the utmost affluence, who are compelled to resort to this kind of diet as often as winter comes round. That it is not unwholesome, appears from the fact that (excepting in St. Petersburg) the death rate is no higher in the towns inhabited by people using this food than in the average of towns in France ; and that the meat loses none of its flavor after months of freezing is admitted by all travellers. It will not be over the mark to say that twelve millions of people in the Northern Hemisphere consume one million tons of frozen food during the winter months. This does not include the frozen meat consumed in England.

An Undescribed Disease of Infants.—Dr. Riga (*Movimento Med. Chir.*) has observed a pernicious disease of the mucous membrane of the child's mouth. It consists of the formation of a false membrane between the end of the tongue and the frænum. The membrane is round and small. Children in whose mouth this appears lose strength rapidly, refuse to nurse, and ninety per cent. of them die. The disease has been observed only in summer, and is always associated with intestinal catarrh. It lasts from two to eight weeks. It is found in children only during the first dentition; it is not contagious, but appears to be infectious. In the Terra di Levoro the disease has been endemic for sixty or seventy years. During the past decade there has been no diphtheria in that region. No scientific study of the disease or membrane has yet been made.—*Phys. and Surg.*

The Most Effectual Engine Discs which we have ever used are made of sand-paper. To be sure they are short-lived, but they are cheap and easily prepared. Use Baeder, Adamson & Co.'s, No. 1 and No. 2 sand-paper, coat the smooth side with gum shellac, cut out the discs with a gun-wad punch, make a hole in the centre with a rubber-dam punch and mount on a screw-head mandrel. For finishing gold fillings and for separating teeth we have found them unequalled, and if any of our readers have never tried them we advise them to be strangers to them no longer.

The Causes of Malaria.—Signor Torelli, who has recently published a map illustrating the prevalence of malaria in Italy, holds that the two principal causes of malaria are the spread of railways and the destruction of forests. Railway embankments interfere with natural drainage, and the destruction of forests causes long periods of drought, during which the earth becomes dry and porous as a sponge, so that when the rain does fall, instead of running off from the surface it is absorbed by the soil, which thereupon remains moist and gives forth noxious vapors for a long period.

Professor Koch's Discovery Disputed.—At a meeting of the New-Orleans Pathological Society, November 20, the President, Dr. H. D. Schmidt, made an important microscopic demonstration to disprove the reported discovery of Professor Koch, in Berlin, as to the bacilli of tuberculosis. Dr. Schmidt claimed to demonstrate that the bacilli thought by Dr. Koch to be the cause of tubercular consumption were simply fatty crystals. Dr. Schmidt, whose researches have been long and minute, is confident that Dr. Koch is in error.

Dr. J. Williston Wright, Professor of Surgery in the University Medical College, who has recently been appointed one of the visiting surgeons to Bellevue Hospital, a few weeks ago successfully performed the operation for removal of the kidney. Extirpation has been performed not over one hundred and twenty times and, of this number, only about fifty patients made a good recovery. Dr. Wright's patient is of the latter category.

Boston Health Ordinance.—By an order adopted November 22, the Boston Board of Health forbade public funerals over the remains of persons who have died of smallpox, scarlet fever, diphtheria, or typhus fever, unless the written permission of the board is first obtained. Bodies of such persons must be placed in tight coffins and may not be exposed to view.

Quinine in Abortion.—The question of the production of abortion by large doses of quinine is still a mooted one. Manson, in the *Maryland Medical Journal,* regards its administration as a preventive of it, his views being based on a forty years' experience in a malarial district.

Dr. Holmes' Successor.—Dr. Thomas Dwight, former Professor of Anatomy at Bowdoin College, and grandson of Dr. John C. Warren, the predecessor of Dr. Holmes, will complete the course of lectures on anatomy at the Harvard Medical School this winter.

Professor Bryant, of Bellevue Hospital Medical College, has been appointed Surgeon-General of the State of New York by Governor-elect Cleveland.

Beginning with the number for Saturday, January 6, *The New York Medical Journal* becomes a weekly instead of a monthly.

